# The Relationship Between Severity of Epilepsy and Sleep Disorder in Epileptic Children

**Published:** 2019

**Authors:** Maryam DEHGHANI, Afshin FAYYAZI, Fatemeh CHERAGHI, Hamideh HAKIMI, Somayeh MOSAZADEH, Saeideh ALMASI

**Affiliations:** 1 Department of Pediatrics Nursing, School of Nahavand Paramedical, Hamadan University of Medical Sciences, Hamadan, Iran; 2 Hearing Disorders Research Center, hamadan university of medical sciences, hamedan, Iran; 3 Chronic Diseases (Home Care) Research Center, Hamadan University of Medical Sciences, Hamadan, Iran.; 4 Department of Nursing, Islamic Azad University of Lahijan, Lahijan, Iran; 5 Department of nursing student research committee, Shiraz University of Medical Sciences, Shiraz, Iran; 6 Department of Pediatric Nursing, Lorestan University of Medical Sciences, Khorramabad, Iran

**Keywords:** Sleep, Sleep disturbance, Epilepsy, Children

## Abstract

**Objective:**

Sleep disturbances are one of the most common behavioral problems in childhood. Sleep problems have an even greater prevalence in children with epilepsy and are one of the most common comorbid conditions in childhood epilepsy.

**Materials & Methods:**

This descriptive-correlation study with the general goal of determining the effects of epilepsy on sleep habits of epileptic children was conducted in Hamadan, western Iran, in 2014. Sampling was done using convenience sampling techniques. Data were collected using the Early Childhood Epilepsy Severity Scale (E-Chess) and Children’s Sleep Habits Questionnaire (CSHQ) and analyzed using SPSS and descriptive and inferential statistics.

**Results:**

The mean score of sleep habits was 55/08±6/71. Bedtime resistance (12/14±2/93), parasomnias (11/02±1/84) and sleep anxiety (8/29±2/46) were the most frequent sleep disorders in the studied sample. Based on Pearson’s r, there were significant positive bidirectional relationships between bedtime resistance (r_s_ =0.129, *P*<0.019), parasomnias (r_s_ =0.298, *P*<0.005), sleep-disordered breathing (r_s_ =0.295, *P*<0.005), CSHQ total score (r_s_ =0.144, *P*<0.022) on the one hand, and children’s epilepsy severity on the other.

**Conclusion:**

Sleep problems should not be overlooked, and a comprehensive review of the sleep habits of this group of patients should be conducted.

## Introduction

Seizures are short-term intermittent changes in movement or behavioral activities caused by abnormal electrical activities of the brain. Less than 1/3 of seizures in children are epileptic (1). Almost 1% of children suffer from epilepsy (2, 3). Epilepsy is prevalent in developing countries including Iran where it is highly prevalent (1.8%) (4). 

It is one of the commonest chronic neurological disorders and can negatively affect patients’ lives (5). Epileptic children, compared with healthy ones, usually experience many behavioral and mental problems (5). This group of children, compared with their healthy peers, have more behavioral, emotional, and communicative issues which bring about dysfunctions in their performance and social abilities in society and school community (6). However, one of the prevalent yet commonly neglected behavioral problems of epileptic children is sleep disorders (5). Sufficient sleep and rest is a primary and basic need for survival and health in all age groups. Sleep quality, and not merely sleep quantity, is essential for desirable performance (7) and recovery from illness (8). People have their own habits and behavioral pattern of sleep, but it is a fact that following a regular schedule and having a good sleep habit facilitate the process of sleep and bring about a sense of rest upon waking. Bad sleep habits decrease the quality of sleep and cause physical and psychological-behavioral disorders (6) such as fatigue, lethargy, depression, negative attitudes, and disturbances in everyday schedule (9). 

There is a complex, bidirectional relationship between sleep and epilepsy so that each variable can influence the other (5, 10). Sleep-wake cycle relates to important changes in electrical activity of brain and also hormonal activities. Therefore, seizures and sleep-wake cycles are completely interrelated. Some people experience seizure while asleep or sleep-deprived, or upon waking up. In others, seizures happen during day or night (11). Sleep phases alter the morphology of epileptiform discharges in a number of childhood epilepsy syndromes (12). Sleep has two main phases: 1. Non-rapid eye movement (NREM), and 2. Rapid eye movements (REM). These two phases are caused by activities in various parts of the brain. In deep sleep, there are few body movements and breathing is regular. Most seizures, especially generalized seizures, probably occur in this phase. Non-deep sleep is characterized by eye movements; hypnic jerks in face, arms, and legs; and irregular quickened breathing. This phase is known as active sleep and mostly occurs while dreaming. Non-deep sleep can last from several minutes to half an hour. Most partial seizures apparently take place in this phase (11). 

Disorders such as clinical sleep deprivation can stimulate seizure (13). Sleep and sleep deprivation have important roles in the onset of epilepsy, appearance of epileptic waves in EEG, idiopathic and symptomatic epilepsy. This is especially important for children because of the role of time of seizures in forecasting by patients (14). Treatable sleep disorders such as obstructive sleep apnea (15), restless legs syndrome, and periodic limb movement, mistaken by parents or physicians as relapse of seizures, may happen in epileptic children. These disorders disrupt sleep and cause excessive daytime sleepiness which, in turn, prevents successful seizure control (7). In addition, sleep in epileptic children may be affected by severity of epilepsy. For example, sleep disorders are more intense in children with generalized epilepsy and drug-resistant epilepsy (16). Mean scores of Children’s Sleep Habits Questionnaire (CSHQ) and subscores of parasomnias, night waking, sleep duration, daytime sleepiness, sleep onset delay, and bedtime resistance were higher for epileptic children in comparison with healthy children. Children with epilepsy had the highest degrees of sleep disorder (2). Although epileptic and healthy children had similar sleep patterns and mean scores of CSHQ were high for both groups, these scores were higher for epileptics (except for subscores of sleep duration and daytime sleepiness) and they were, therefore, suffering from more severe sleep disorders (5). Number, type, and time of seizures may disrupt sleep-wake cycle, and anti-epileptic drugs (AEDs) affect the normal structure of sleep and reduce sleep sufficiency (7). The severity of epilepsy (including number and type of seizures, number of AEDs and their side-effects) was the only predictor of sleep disorders in epileptic children (12). Children with generalized seizures had more incidences of sleep disorder compared with those with focal seizures (16). Children and adolescents with drug-resistant epilepsy, polytherapy, night seizures, and delayed growth show bad sleep habits, behaviors, and quality which may, in turn, negatively affect seizure control (16, 17). However, no statistically significant difference was found between types of seizures and sleep disorders (5).

 Considering all this, since epilepsy is not merely a physiologic disease of central nervous system, and has numerous mental and psychological effects on the lives of children and their parents and imposes the heavy burden of, among others, economic problems on them, all medical team members including nurses identify predisposing and aggravating factors such as sleep quality and habits upon meeting an epileptic child, and change the environment in order to decrease or prevent seizures. In addition to identification of predisposing and aggravating factors, one of the most important duties of physicians and nurses is to train patients and their families in controlling the disease. Training can enhance the understanding of health and disease conditions on the part of patients and their families, which can in turn increase motivation and cooperation in deciding to change lifestyles and adopting healthy behaviors. Although clinical relations between sleep and epilepsy have already been studied, few such studies have been conducted on children. The researchers decided to investigate the relationship between sleep habits and seizure in children with epilepsy referring to Imam Khomeini Outpatient Clinic in Hamedan, Iran, in order to take steps for enhancement of child care, focusing on provision of disease control training for families. 

The present study had the following aims: determination of 1) sleep habits, 2) epilepsy severity, 3) the relation between sleep habits and epilepsy severity, and 4) the relation between sleep habits and type of AEDs. 

## Materials & Methods

**Inclusion and exclusion criteria**

This descriptive-correlation study with the general goal of determining the effects of epilepsy on sleep habits of epileptic children was conducted in Hamedan, western Iran, in 2014. Research population consisted of all epileptic children of 1-12 yr of age. Sleep quality and habits of children are affected by diagnostic and treatment procedures during hospitalization; thus, Imam Khomeini Outpatient Clinic was selected as the research site. Sample size was determined as 100 patients (2), with *P*=0.049 and a confidence interval of 90%, and by considering the probability of attrition. Sampling was done using convenience sampling techniques. 

Inclusion criteria were as follows: 1) children’s age must be in the range of 1 to 12 yr; 2) they should not be hospitalized during the course of study; 3) epilepsy must be diagnosed by a consulting physician; 4) they should not have used sedatives except for AEDs in the past 3 months; 5) they should not be afflicted by chronic respiratory diseases or sleep-related respiratory disorders; (6) they should not have other neurological disorders; and (7) parents or caretakers must be literate (2, 5).

**Questionnaires**

a) demographic information questionnaire, including 18 questions on: age, sex, duration of epilepsy, age of first seizure, type of medication taken, duration of taking medication, family history of epilepsy, parents’ education and occupation, etc. 

b) Children’s Sleep Habits Questionnaire (CSHQ): As a parent-reported Questionnaire, CSHQ includes 33 items in the form of 3-point Likert scales on eight subscales of: Bedtime Resistance (6 items), Sleep Aanxiety (4), Parasomnias (7), Sleep Disordered Breathing (3), Night Waking (3), Daytime Sleepiness (8), Sleep Duration (3), and Sleep Onset Delay (1). It is a standard questionnaire designed and validated with the purpose of evaluating children’s sleep habits, with Cronbach’s alpha of 0.68-0.78 and test-retest reliability of 0.62-0.79 (18). The Cronbach’s alpha of 0.78 was reported on 20 Iran children of 7-11 yr of age (8). In the present study, Cronbach’s alpha for the entire scale was 0.79. Parents were asked to think about their children’s sleep pattern in the past weeks, and choose “most of the time” (3 scores) for 5-7 repetitions of a habit, “sometimes” (2 scores) for 2-4, and “rarely” (1 score) for 0-1. There are 2 items (Item 5 and 8) that are common to the bedtime resistance and sleep anxiety subscales. A total score can be obtained by summing up the scores of the 33 items and the score range was 33- 99. Subscale’s scores can be obtained by summing up their respective items. The higher the total score (≥41), the child has more sleep problems (18). 

c) The Early Childhood Epilepsy Severity Scale (E-Chess): This questionnaire includes 6 questions on seizure frequency, time period over which seizures occurred, number of seizure types, occurrence and duration of status epilepticus, number of anticonvulsant medications used, and response to treatment, and was designed and validated. Items on frequency of seizures, time period over which seizures occurred, number of seizure types, and response to treatment were scored 1-3, and items on status epilepticus, occurrence, and duration of episode and number of anticonvulsant medications used were scored 0-3, and the total score was calculated by adding these two. The higher the total score, the more severe epilepsy (19).

**Ethics approval**

Letters of introduction were obtained from the Vice-presidency of Research at Hamedan University of Medical Sciences and Chronic Disease Research Center, and written consent forms were received from parents by visiting Imam Khomeini Outpatient Clinic. The researcher filled the questionnaires by conducting interviews and referring to patients’ medical history.

**Statistical analysis**

Data were analyzed by SPSS ver. 20 (Chicago, IL, USA) using descriptive (frequency, percentage, mean, SD) and inferential (Pearson’s r and independent-samples *t*-test) statistics. Alpha of 0.05 was considered as the significant level.

## Results

**Demographic data**

Overall, 49 (50%) boys and 49 (50%) girls with mean age and SD of 5.69±2.76 yr participated in the study. Most of them (83.7%) slept in the same room as their parents, and only 16.3% had separate bedrooms. Mean duration of sleep was 9.43±1.52 h at nights and 1.57±1.01 h during daytime. Mean age of first seizures was 3.20±2.88 yr and the duration of affliction with epilepsy was 2.89±2.4 yr. In 47.3% of children seizures happened in daytime, in 14% at nights, and in 38.7% there was no fixed time. Mean number of seizures was 4.17±5.01 a year. Mean duration of taking AEDs was 2.89±2.24 yr, and most of the children (44.3%) were prescribed sodium valproate. Among them, 49.5% had a family history of epilepsy. Mean age of fathers was 37.38±7.57 and that of mothers was 32.25±7.32 yr. The majority of mothers (43.9%) had primary education, and only 8.2% had attended universities. Most of them (97%) did not have a job (were homemakers). The majority of fathers (45.4%) had primary education, and only 9.3% had attended universities. Most of them (80.4%) were self-employed (Table1).

**Table 1:** Demographic and clinical characteristics of the samples

Children	Number (%) of patients and Mean ± SD
Age, years, M (SD)	5.69(2.76)
Sex	
Male Female	49(50) 49(50)
Duration of sleep, hours, M (SD)	
Night Daytime	9.43(1.52) 1.57(1.01)
Age of first seizures, years, M (SD)	3.20(2.88)
Duration of epilepsy, years, mean (SD)	2.89(2.40)
Seizure time	
Daytime predominant Nocturnal predominant No predominant timing in sleep cycle	44(44.90) 13(13.26) 41(41.84)
Number of seizures, years, M (SD)	4.17(5.01)
Family history of epilepsy	
Yes No	48(48.98) 50(51.02)
Medication used	
Valproate Carbamazepine Topiramate Clonazepam Gabapentin Clobazam Lamotrigine Phenobarbital Phenytoin Primidone Others	43(43.87) 28(28.57) 7(7.14) 3(3.06) 1(1.02) 3(3.06) 6(6.12) 18(18.36) 7(7.14) 24(24.48) 19(19.39)

**Table 2:** Children’s Sleep Habits Questionnaire total and subscale scores

CSHQ subscales	Mean ± SD
Bedtime resistance	12.14 (2.93)
Sleep onset delay	1.47 (0.77)
Sleep duration	4.29 (1.65)
Sleep anxiety	8.29 (2.46)
Night waking	5.04 (1.55)
Parasomnias	11.02 (1.84)
Sleep-disordered breathing	4.06 (1.22)
Daytime sleepiness	13.06 (3.13)
Total score	55.08 (6.71)

**Sleep questionnaire**

Mean and SD were 55.08±6.71 for total score of CSHQ, 12.14±2.93 for bedtime resistance, 8.29±2.46 for sleep anxiety, 11.02±1.84 for parasomnias, 4.06±1.22 for sleep-disordered breathing, 5.04±1.55 for night waking, 13.06±3.13 for daytime sleepiness, 4.29±1.65 for sleep duration and 1.47±0.77, for sleep onset delay (Table 2).

In “bedtime resistance” section, items “The child needs to be near his/her parents in order to fall sleep” (62.2%) and “The child is afraid of sleeping alone.” (51%) were the most frequent ones (Usually). 

Based on parents’ reports, in “sleep onset delay” section, most of the children (69.4%) could not fall sleep within the first 20 min of going to bed.

 Under “sleep duration”, most of them (74.5%) rarely received enough sleep. Under “sleep anxiety”, items “The child needs to be near his/her parents in order to fall sleep.” (62.2%) and “The child is afraid of sleeping alone.” (51%) were the most frequent ones (Usually).

 Under “night waking”, most of them (64.3%) rarely woke up during the night or went to others’ bedrooms, and only 20.4% of the children woke up more than once.

 In “parasomnias” section, 93.9% of children rarely sleepwalked, while 78.6% usually were restless and moved a lot during sleep.

 In "sleep-disordered breathing” section, most of the children (86.7%) rarely experienced dyspnea. 

Under “daytime sleepiness”, 74.5% of them rarely woke up in the morning by themselves, and 50.5% were usually drowsy or napped in the car.

**Early Childhood Epilepsy Severity Scale and Associations between sleep parameters and epilepsy**

Another goal of this study was determining epilepsy severity and its relation with sleep habits. Mean and SD of epilepsy severity in children were 9.38±1.68. According to parents’ answers to “frequency of seizure” question, in the onset of epilepsy and before seizures were controlled by antiepileptic drugs most of the children (59.8%) had weekly seizures and only 25.8% experienced daily seizures. After start treatment and when seizures were controlled by antiepileptic drugs, children had at least once every month 9.2%, at least once every year 77.6% and less than once every year 13.2%. The most frequent answer to “time period over which seizures occurred” was more than 6 months (65.3%). The most frequent “number of seizure types” was only one seizure type (55.6%). For 96.6% of children, there has been no “occurrence of status epilepticus”, while 3.1% had experienced it for the duration of 30 min to 1 h. The “number of anticonvulsant medications used” was 1 or 2 for 84.7% of children. As for "response to treatment”, seizures were almost stopped in 71.4% of children, while no improvement was observed in 16.3%. 

Based on Pearson’s r, there were significant positive bidirectional relationships between bedtime resistance (r_s_ =0.129, *P*<0.019), parasomnias (r_s_ =0.298, *P*<0.005), sleep-disordered breathing (r_s_ =0.295, *P*<0.005), CSHQ total score (r_s_ =0.144, *P*<0.022) on the one hand, and children’s epilepsy severity on the other.

As for the fourth goal of the study, that was, determining the relationship between sleep habits and type of AEDs; independent-samples t-tests were run. Results of comparing children who took sodium valproate with those who did not, on subscores of “night wakings” (*P*<0.011) and total score of CSHQ (*P*<0.120) were significant; these scores were higher for children who took this medication. Moreover, a significant difference was observed between children who took phenytoin and those who did not, on subscores of “nigh wakings” (*P*<0.13); these scores were higher for children who took this medication.

## Discussion

As mean score and SD of CSHQ were 55.08±6.17 in this study, most of the children had bad sleep habits and low sleep quality and suffered from many sleep disorders. Bedtime resistance, parasomnias and sleep anxiety were the most frequent sleep disorders in the studied sample. In line with the present study, 48.25±8.912 was reported as the mean score of CSHQ, and “parasomnias”, “night waking”, “sleep duration”, “daytime sleepiness”, “sleep onset delay”, and “bedtime resistance” as more prevalent among epileptic children compared with healthy ones. However, no significant difference was observed between the two groups on subscores of “sleep-disordered breathing” and “sleep anxiety” (2). 77.8% of children with epilepsy slept in their own rooms. In another study, “Mean total score of Sleep behavior questionnaire (SBQC) was 58.76±17.72. 50% had parent/child interaction issues characterized by falling asleep again with parental presence, 39% had sleep fragmentation with awakenings up to 3-4 times per night, 61% had parasomnia, and 44% showed daytime sleepiness characterized by sleepiness while sitting or studying and watching TV” (20). Overall, 60% of children with epilepsy slept in a room separate from their parents, while in the present study only 16.3% had separate bedrooms. Similar to our study, epileptic children had worse sleep habits compared with their healthy peers; for instance, they needed to be put to bed by their parents, took more than 30 min to fall asleep, had an afternoon nap, expressed fear of the dark after being put to bed for the night, awoke in distress/worry from a dream, kept a nightlight on while sleeping, and called for a parent during the night (16). Although the sleep patterns of children with and without epilepsy were similar, and mean and SD of CSHQ scores were high for both groups (44.10±6.43 for healthy children, and 48.89±6.83 for children with epilepsy), results of one-way ANOVA revealed that total CSHQ score and all its subscales (except for “sleep duration” and “daytime sleepiness”) were much higher for epileptic children, and they had worse sleep habits than their peers (5). Nevertheless, no significant difference was found between mean score and SD of CSHQ of healthy (43.47±6.35) and epileptic (43.09±5.41) children (21). This study is in line with previous cases in that despite differences between sleep habits of children from various countries, it showed worse sleep habits, poorer sleep quality, and more sleep disorders for children with epilepsy. One of the reasons for this phenomenon may be the fact that parents worry their children might experience nocturnal seizures and as a result, check on them frequently during the night, which in turn brings about sleep disruption, night wakings, insufficient sleep, anxiety, and a rise in reporting sleep disorders by parents (5). Mean score and SD of CSHQ reported here was higher compared with other studies (2, 5, 20), which may be due to cultural differences, child discipline rules in families of different societies, and strategies adopted by parents in order to regulate children's sleep pattern.

Another goal of this study was determining epilepsy severity and its relation with sleep habits. In line with this study, reported mean and SD of children's epilepsy severity to be 10.47±3.82; and 25.7% had severe epilepsy with scores of ≥14. 64.2% of children experienced at least one seizure within the previous month, and 36.8% had daily seizures. Mean numbers of current AEDs prescribed were 2.16±1.27, and 90.5% took at least one medication (2). Likewise, 55.5% of children had experienced at least one seizure in the previous month. 46.5% took one, 50% took two, and 14.7% took no AEDs (22). Moreover, 89% of children with epilepsy were taking AEDs; 62% on monotherapy and 27% on polytherapy. In this study, 41% of children had not experienced seizures in the over past 6 months, 29% had experienced at least one seizure in the over past 6 months; 11% at least once every month; 11% at least once every week; and 8% at least once every day(5). Mean score of E-Chess were 6±1.18, and the score was high in 52.2% of children (12). Results of this study are similar to those of previous studies, except for some sections of E-Chess, in which children received high scores.

 Here, a significant positive bidirectional relationship was observed between sections of “bedtime resistance”, “parasomnias”, “sleep-disordered breathing”, and CSHQ total score on the one hand, and children’s epilepsy severity on the other. There was a complex, bidirectional relationship between sleep and epilepsy so that each variable can influence the other (5). Similarly, a significant positive bidirectional relationship was found between epilepsy severity and CSHQ total score. Among CSHQ subscales, only “parasomnias” and “sleep wakings” had significant relations with epilepsy severity (2). The severity of epilepsy (including number and type of seizures, number of AEDs and their side-effects) was the only predictor of sleep disorders in epileptic children (12). In this section, we refer to studies assessed epilepsy severity using different measures such as type of seizures, number of types of seizures, and number of AEDs, and have checked their relationship with children's sleep habits. Overall, 48% of children took no medication, 50% took one, and 2% took two. A significant relationship was revealed between number of AEDs and level of parasomnia symptoms, and also between type of seizures and syndrome to sleep fragmentation. “Children with partial seizures with secondary or symptomatic/cryptogenic syndromes had more difficulty with sleep fragmentation than children with generalized tonic-clonic seizures or idiopathic syndromes” (13). About, 75% of epileptic children took one, and 25% took more than one AEDs; the former had better sleep habits compared with the latter (16). Children and adolescents with drug-resistant epilepsy, polytherapy, night seizures, and delayed growth show bad sleep habits, behaviors, and quality which may, in turn, negatively affect seizure control (16, 23). Nevertheless, although 89% of children took AEDs (62% one, and 27% more than one); there was no significant difference between the number of medications and sleep problems (5).

There was a significant difference between children who took sodium valproate and those who did not, on subscale of “night waking’s” and total score of CSHQ; moreover, there was a significant difference between children who took phenytoin and those who did not, on subscale of “night waking’s”. Side-effects of AEDs are among the main obstacles of finding the right dose for controlling seizures. They include fatigue, drowsiness, imbalance, memory problems, and attention disorders. In addition, AEDs affect sleep duration, sleep latency, and sleep architecture (24-26). Sodium valproate is an AED. This medication may increase NREM, decrease REM and excessive sleepiness (26). Termination of sodium valproate after long-term treatment can lead to a significant reduction in sleep duration in children older than 6 yr of age (27). Similar to this study, 55% of children were prescribed sodium valproate. Among AEDs, significant differences were observed between the use of sodium valproate and total score of CSHQ (50.89±7.49 and 46.39±4.96, *P*<0.008), and subscale of “night wakings” (4.09±1.31 and 3.43±0.74, *P*<0.022), and between clobazam and subscale of “sleep-disordered breathing” (5.00±1.59 and 3.64±0.91, *P*<0.043) (5). Monotherapy with the smallest dose of an effective AED, minimum sleep disorders, and paying attention to the right time of drug prescription may improve sleep and quality of life in patients with epilepsy.

This study had a number of limitations. Sleep quality and habits were assessed using parents’ reports; other studies can use tools such as polysomnography for the diagnosis of sleep disorders and compare sleep habits of epileptic children with those of healthy children or children with other chronic diseases.

**In Conclusion**, epilepsy is one of the commonest chronic neurological disorders and negatively affects children's cognitive, social, and emotional performance. Although sleep disorders are common in ill children, they are more so in epileptic ones and are usually neglected. Sleep disorders expose children to mood, cognitive, and behavioral disorders and also seriously affects their physical health. Assessment of sleep habits must be a part of general care of epileptic children. Moreover, other parameters such types of seizures, sleep-related respiratory disorders, and types of AEDs must be evaluated. Thus, all medical team members including nurses identify predisposing and aggravating factors such as sleep quality and habits upon meeting an epileptic child and change the environment in order to decrease or prevent seizures. In addition, information regarding strategies of healthy sleep is provided for families, especially those with little education or highly stressed.
